# Research progress on the mechanisms of mucus retention after mycoplasma pneumoniae infection

**DOI:** 10.3389/fcimb.2026.1751803

**Published:** 2026-03-24

**Authors:** Xinran Li, Yucong Ma, Shipeng Cao, Wei Wu, Li Liu

**Affiliations:** Department of Pediatric Respiratory, Children’s Medical Center, First Affiliated Hospital of Jilin University, Changchun, China

**Keywords:** immune-inflammatory response, MUC5AC, mucus hypersecretion, mucus retention, mycoplasma pneumoniae, treatment strategies

## Abstract

Mycoplasma pneumoniae (MP) is a significant pathogen causing community-acquired pneumonia (CAP) in children, often complicated by bronchial mucus plugs (BMPs), which can lead to atelectasis, respiratory failure, and other severe outcomes. This narrative review comprehensively discusses the molecular and cellular mechanisms underlying mucus retention post-MP infection, focusing on pathogen-host interactions, cytokine release, and activation of inflammatory signaling pathways that drive abnormal physicochemical changes in mucus. MP adheres to respiratory epithelial cells, inducing oxidative stress and cellular damage, while activating pathways such as NF-κB and MAPK to promote excessive mucin production. The host immune-inflammatory response—including neutrophil extracellular trap formation, Th1/Th2 imbalance, and release of cytokines like IL-4, IL-13, and TNF-α—further exacerbates mucus hypersecretion. Concurrently, physicochemical alterations in mucus combined with impaired ciliary clearance facilitate the transition of mucus from a sol to a gel state, ultimately causing mucus retention. The review also summarizes targeted therapeutic strategies, including macrolides, mucolytics, anti-inflammatory agents, traditional Chinese medicine (TCM) Therapies, and bronchoscopic interventions, to inform clinical management. Future research should prioritize elucidating drug-resistant mechanisms in macrolide-resistant mycoplasma pneumoniae (MRMP) and developing precision therapies to improve patient prognosis.

## 1 Introduction

Mycoplasma pneumoniae (MP) is a globally prevalent respiratory pathogen capable of infecting both the upper and lower respiratory tracts. It induces endemic and epidemic infections in both children and adults, with children being particularly susceptible. Among pediatric populations, MP is responsible for 10% to 40% of community-acquired pneumonia (CAP) cases, and this proportion may surge to 20% to 70% during pandemic periods (Liu et al., 2024). In China, it boasts the highest detection rate among atypical pathogens in children aged 5 to 7 years (Song et al., 2023). Although most patients follow a self-limiting clinical course, some cases—especially in children—often progress to severe pneumonia.

Pneumonia, particularly cases induced by specific pathogens such as MP, respiratory syncytial virus (RSV), or adenovirus, is frequently accompanied by airway mucus hypersecretion, leading to mucus retention—a critical pathological feature that influences disease severity and prognosis. Airway mucus retention represents a core pathological characteristic of various respiratory disorders, referring to the abnormal accumulation and impaired clearance of mucus within the airways (Hill et al., 2022). It is not an isolated symptom but rather a comprehensive outcome of a series of complex processes involving mucus production, secretion, altered physicochemical properties, and compromised clearance mechanisms. This condition directly contributes to airflow obstruction, recurrent infections, and progressive lung injury (Ramsey et al., 2020).

Previous studies have demonstrated that infections with multiple respiratory pathogens can induce mucus hypersecretion. Bacterial components such as lipopolysaccharide (LPS) are key substances driving excessive airway mucus secretion (Benedé-Ubieto et al., 2024). LPS can directly stimulate human airway epithelial cells, increasing the secretion of mucin MUC5AC and the production of pro-inflammatory cytokines (IL-1β, IL-6, TNF-α). LPS promotes heightened mucus secretion through the formation of neutrophil extracellular traps (NETs) and by modulating the TLR4/NF-κB signaling pathway. Conversely, degrading NETs and inhibiting the TLR4/NF-κB pathway can reduce the release of pro-inflammatory factors from macrophages, thereby alleviating LPS-induced airway inflammation and mucus hypersecretion (Zou et al., 2018). Pyocyanin, via suppression of the transcription factor FOXA2, leads to goblet cell hyperplasia and metaplasia, further promoting increased expression of the MUC5B mucin gene and enhanced mucus hypersecretion (Hao et al., 2012). Clinical research has shown that integrin β4 (ITGB4) in airway epithelial cells (AECs) of children following RSV infection plays a key role in regulating airway mucus secretion. ITGB4 can reduce airway mucin MUC5AC levels by inhibiting the EGFR/ERK/c-Jun pathway (Du et al., 2022). Additionally, human rhinoviruses (HRVs) infection can induce airway mucus synthesis and secretion through an NF-κB-dependent MMP–TGF-α–EGFR–MEK/ERK–Sp1 signaling cascade (Hewson et al., 2010).

Airway hypersecretion leading to mucus retention represents a key pathological feature of pneumonia, with severe cases potentially resulting in the formation of bronchial mucus plugs (BMPs). Among children with Mycoplasma pneumoniae pneumonia (MPP), bronchoscopy can reveal airway lumen obstruction by secretions composed of mucins, and some cases exhibit characteristic changes of plastic bronchitis (PB) (Ma et al., 2022). However, due to the lack of specific early clinical manifestations and imaging features, BMPs are prone to misdiagnosis or missed diagnosis in the early stage of the disease. When symptoms become obvious, severe complications have often already occurred. Therefore, in-depth research on the mechanism of mucus retention after MP infection is of great significance for early diagnosis, intervention, and improvement of prognosis.

In recent years, with the in-depth development of molecular biology and immunology research, significant progress has been made in understanding the mechanism of airway mucus hypersecretion induced by MP. Studies have shown that this process involves the complex interaction of multiple factors, including direct pathogen-induced injury, excessive activation of the host immune response, alterations in mucus composition, and impaired ciliary clearance function (Liang et al., 2026). In particular, the emergence of macrolide-resistant mycoplasma pneumoniae (MRMP) has further complicated this issue. Research has confirmed that MRMP is an independent risk factor for bronchial mucus plug formation in children, and the risk of mucus plug development in children infected with MRMP is higher than that in those infected with macrolide-susceptible strains (Xie et al., 2025).

This review aims to systematically summarize the molecular and cellular mechanisms underlying mucus retention following MP infection, analyze the key pathogenic factors and clinical characteristics, and provide a theoretical framework for future research directions and therapeutic strategies. By integrating multi-dimensional evidence such as pathogen virulence factors and host immune responses, it offers novel insights for clinical treatment.

## 2 Mechanism of mucus secretion in the normal airway

Airway mucus in healthy individuals consists of 97% water and 3% solids, including mucins, non-mucins, salts, lipids, and cellular debris. The main mucins in healthy mucus are MUC5B and a small amount of MUC5AC, both of which are crucial for lung homeostasis, host defense, and effective mucus transport (Roy et al., 2014; Livraghi-Butrico et al., 2017; Hancock et al., 2018). Mucin concentration is essential for efficient mucus transport; an abnormal increase in mucin concentration affects mucus viscoelasticity and impairs mucociliary clearance (Tajiri et al., 2022). Specifically, MUC5AC is produced by goblet cells on the epithelial surface, while MUC5B is mainly synthesized by mucus cells in submucosal glands. The ratio of these two mucins is associated with human health status—for example, MUC5AC is the predominant mucin in patients with asthma, whereas MUC5B is the major mucin in healthy individuals (Wallace et al., 2021; Hill et al., 2022; Tajiri et al., 2022). Mucus serves as a key defense mechanism against harmful environmental exposures, as it can trap and eliminate inhaled particles while keeping the airway surface moist. Under normal conditions, the volume of airway mucus is relatively small. However, airway infection by pathogens can induce mucosal cell metaplasia and hyperplasia, leading to mucus hypersecretion. Imbalance in mucus not only causes respiratory symptoms but also contributes to disease progression, decreased lung function, and increased mortality (Williams et al., 2006; Tajiri et al., 2022).

## 3 Direct evidence of airway mucus hypersecretion induced by MP infection

Existing studies have clearly confirmed that MP infection can significantly promote airway mucus hypersecretion (Chang et al., 2024). In a study by M. Kraft et al. on MP-induced MUC5AC expression in asthmatic airway epithelial cells, it was first demonstrated in humans that the expression of MUC5AC—the major mucin in human airways—increases following MP infection (Kraft et al., 2008). Further clinical studies have shown that the concentrations of MUC5AC and MUC5B in the bronchoalveolar lavage fluid (BALF) of patients infected with MP are significantly elevated, with a more pronounced increase in those complicated by PB. Given that MUC5AC and MUC5B are the core components of the airway mucus gel, their overexpression provides the material basis for mucus retention (Ma et al., 2022).

Current studies suggest that mucus retention mainly undergoes two key pathological stages. Firstly, the stage of airway mucus hypersecretion, which is mainly caused by direct pathogen invasion and immune regulation disorders, ultimately leading to abnormal expression of mucin genes (MUC5AC/MUC5B) (Hill et al., 2022). Secondly, the stage of altered mucus rheological properties, characterized by changes in mucin molecular conformation and local microenvironmental modifications, which ultimately induce the transformation of mucus from a sol state to a gel state, resulting in mucus retention (Ramasamy et al., 2021). These two stages interact synergistically and collectively constitute the pathological basis for mucus retention.

## 4 Mechanisms of airway hypersecretion induced by MP infection

### 4.1 Direct injury by MP contributes to increased airway mucus secretion

MP causes direct injury by adhering to, colonizing, and invading host cells, and promotes inflammatory responses, thereby inducing airway mucus hypersecretion (Zhang et al., 2022). Adhesion, colonization, and invasion of host cells by MP are the initial stages of MP infection and the prerequisite for all pathological injuries. MP adheres to host cells to consume nutrients and releases virulence factors such as community-acquired respiratory distress syndrome toxin (CARDS Tx), which damages the respiratory epithelium (Hu et al., 2022). CARDS Tx is currently recognized as the most important virulence factor of MP. Studies have shown that CARDS Tx can induce vacuolization, rounding of cell morphology, and disruption of monolayer integrity in host cells, ultimately leading to cell death (Kannan et al., 2011). This toxin can also cause damage to ciliary structures (Parrott et al., 2016). The mucociliary clearance (MCC) system is a key defense mechanism for maintaining airway homeostasis, and it secretes MUC5AC and MUC5B (Wallace et al., 2021; Hill et al., 2022). Furthermore, the MCC system expresses membrane-bound mucins (e.g., MUC1, MUC4, and MUC16), which not only regulate mucus rheological properties but also participate in pathogen recognition and immune regulation (Hill et al., 2022). After MP infection, ciliary structure damage occurs, disrupting this delicate balance. This leads to changes in the physical and chemical properties of mucus and impairment of clearance function, preventing mucus from being promptly cleared by the MCC system and resulting in its retention in the airways.

**Figure 1:**
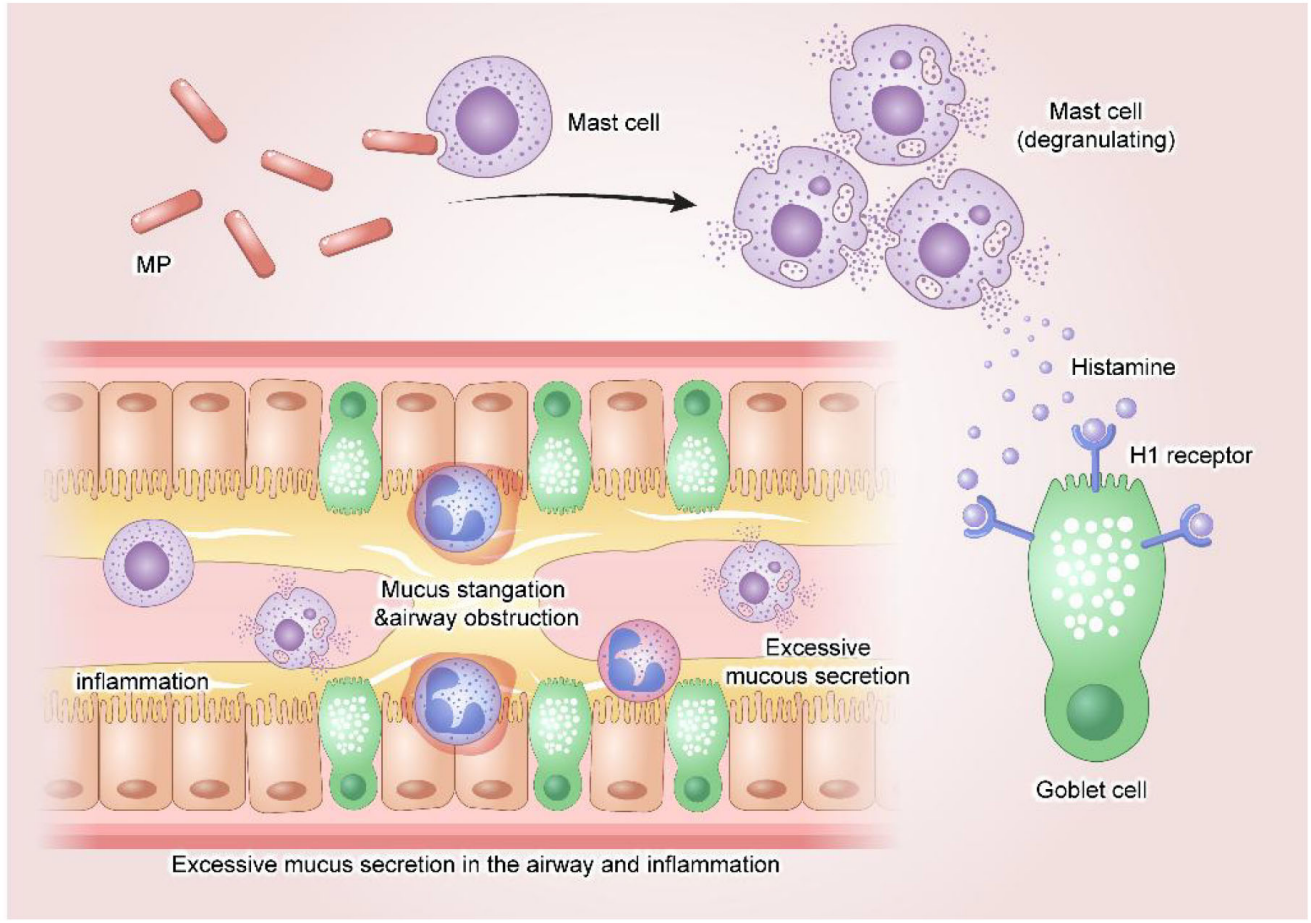
Mycoplasma pneumoniae (MP) causes direct damage by adhering to, colonizing, and invading host cells, triggering an inflammatory response that leads to excessive airway mucus secretion. P1 adhesin, a sensitizing component of MP, plays a critical role in MP-induced mast cell activation: MP activates mast cells through direct contact with sialylated residues on their surface, thereby initiating inflammatory injury. Histamine released from degranulated mast cells binds to H1 receptors on goblet cells, further promoting mucus secretion. (Adobe Illustrator were used in the preparation of the figures).

The P1 adhesin secreted by MP also plays a crucial role. Not only does P1 adhesin mediate the binding of MP to host receptors to exert adhesive effects, but it also participates in the gliding movement of MP on the host cell surface (Hu et al., 2022). Studies have identified P1 adhesin as an allergenic component of MP. It plays a key role in MP-induced mast cell cytokine responses: MP activates mast cells through direct contact with sialylated residues on the mast cell surface, triggering inflammatory damage. Meanwhile, it induces humoral immunity to produce antibodies against P1, including P1-specific IgE, histamine released by mast cell degranulation binds to H1 receptors on the surface of goblet cells, further promoting goblet cell mucus secretion. This excessive mucus production leads to airway obstruction and the development of severe clinical symptoms (Carroll et al., 2002; Ye et al., 2018; Wu et al., 2019; Hu et al., 2022) (**Figure 1**).

### 4.2 Host immune and inflammatory responses induced by MP infection

#### 4.2.1 Activation of immune cells and release of inflammatory mediators

In acute MP infection, neutrophils are recruited and exhibit enhanced activity in pulmonary infection defense. Activated neutrophils release substances such as elastase, matrix metalloproteinases (MMPs), and myeloperoxidase (MPO). Upon external stimulation, neutrophils rapidly generate reactive oxygen species (ROS) and induce oxidative burst. Through the process of NETosis, neutrophils extrude chromatin networks and bind to granular peptides of neutrophil extracellular traps (NETs), releasing decondensed chromatin and granular contents into the extracellular space, which increases the viscoelasticity of airway mucus (Wu et al., 2007; Fu et al., 2021; Pangeni et al., 2023). Additionally, the production of neutrophil elastase can promote MUC5AC expression, leading to mucus hypersecretion and airway obstruction (Lee et al., 2021).

Macrophages play a critical role in MP-induced inflammatory responses. MP infection induces alveolar macrophages to produce IL-23, and IL-23-dependent IL-17/IL-17F further defends against infection by recruiting and activating neutrophils (Wu et al., 2007). Studies have shown that after inflammatory responses occur in lung tissue, infected cells activate multiple signaling pathways and produce a large number of inflammatory mediators, such as macrophage migration inhibitory factor (MIF). As a pleiotropic immunomodulatory cytokine, MIF has potent macrophage activation functions. It can participate in inflammatory responses by activating the ROS pathway and induce the release of cytokines including TNF-α and IL-10 (de Souza et al., 2019). MP infection can trigger asthma-like reactions, and elevated MIF levels have been detected in the BALF, serum, and sputum of asthmatic patients (Li et al., 2021). This suggests that MIF may serve as an early biomarker for mucus plug formation following MP infection.

#### 4.2.2 Leukotriene release induced by MP infection promotes airway mucus secretion

**Figure 2:**
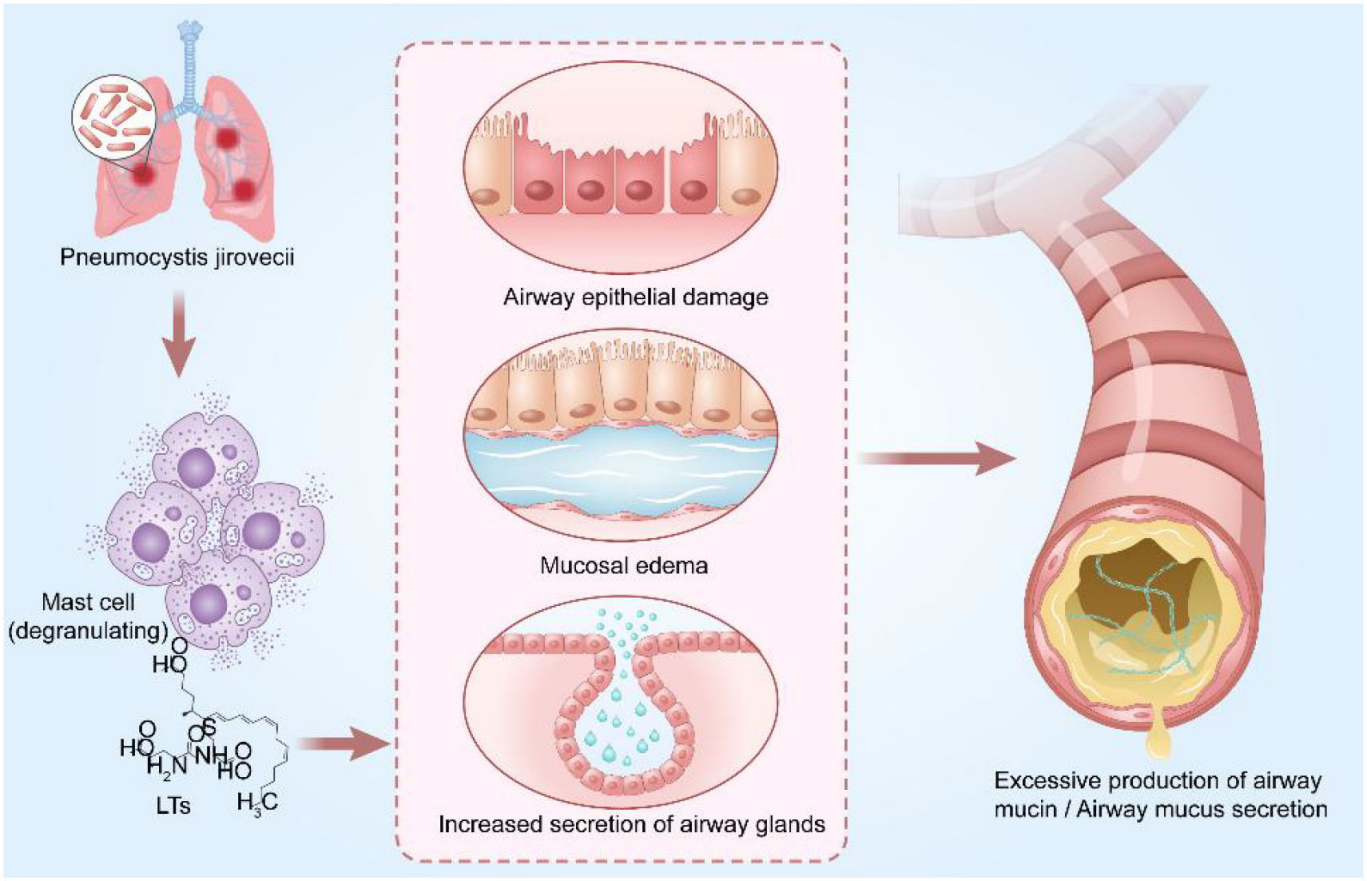
MP infection induces leukotriene release, which promotes airway mucus secretion. Leukotrienes (LTs), primarily released through mast cell degranulation, contribute to airway epithelial damage accompanied by mucosal edema and increased secretion from airway glands, thereby leading to excessive production of airway mucins. (Adobe Illustrator were used in the preparation of the figures).

MP infection can significantly promote the release of leukotrienes (LTs) in the body, a process closely associated with airway mucus hypersecretion and mucus retention. The P1 adhesin of MP has been confirmed to activate mast cells, triggering inflammatory damage (Hu et al., 2022). LTs are mainly released by mast cell degranulation, which can cause airway epithelial destruction accompanied by mucosal edema and increased secretion from airway glands, thereby leading to the overproduction of airway mucins. Among various LTs, cysteinyl leukotrienes (CysLTs) are key mediators of airway allergic diseases. Specifically, LTE4, a type of CysLTs, has been shown to induce airway obstruction and mucosal eosinophil infiltration in humans when administered exogenously (Shirasaki et al., 2015). Studies have found that the level of LTB4 is significantly elevated in patients with MPP, and LTE4 can dose-dependently induce the release of MUC5AC mucin. Additionally, LTs receptor antagonists can effectively inhibit MUC5AC secretion (Bai et al., 2007; Shirasaki et al., 2015; Pan et al., 2024). It can thus be concluded that LTs pathway activation following MP infection promotes airway mucus hypersecretion, providing a theoretical basis for the clinical use of LTs modifiers in the treatment of MP-related airway diseases (**Figure 2**).

#### 4.2.3 Cytokines and Th1/Th2 imbalance

Immune dysregulation induced by MP infection affects airway mucus secretion primarily through imbalance of T cell subsets. Studies have shown that the CARDS Tx produced by MP can activate a positive feedback loop of type 1 immune responses, promoting Th1 cell migration and leading to Th1/Th2 imbalance with a ratio significantly lower than the normal level (Giavina-Bianchi and Kalil, 2016; Yang et al., 2019; Wang et al., 2022). This imbalanced state induces excessive secretion of Th2 cytokines, mainly involving IL-4 and IL-13.

IL-4:

As a key cytokine secreted by Th2 cells, eosinophils, and mast cells, IL-4 plays a central role in immune regulation. This cytokine binds to type II receptors on airway epithelial cells to mediate downstream signaling (Parrott et al., 2016; Iwaszko et al., 2021). Studies have shown that following MP infection, P1 adhesin can induce excessive IL-4 secretion and aberrant differentiation of Th0 cells into Th2 cells, leading to a significant elevation in serum IL-4 levels (Ye et al., 2018; Shen et al., 2024). After activating type II receptors, IL-4 directly induces the phosphorylation of JAK1 and TYK2, which in turn promotes the activation and nuclear translocation of STAT6. It also activates signaling molecules such as STAT3, PI3K, and MAPK (Hao et al., 2014; Li et al., 2018; Iwaszko et al., 2021), ultimately resulting in the overexpression of airway mucins. Additionally, the massive production of IL-4 further induces mast cell proliferation. Histamine released by mast cell degranulation binds to H1 receptors on the surface of goblet cells, thereby further promoting goblet cell mucus secretion (Wu et al., 2019; Husna et al., 2022; Oishi et al., 2025; Sahnoon et al., 2025). These findings confirm the critical regulatory role of IL-4 in airway mucus hypersecretion following MP infection.

**Figure 3:**
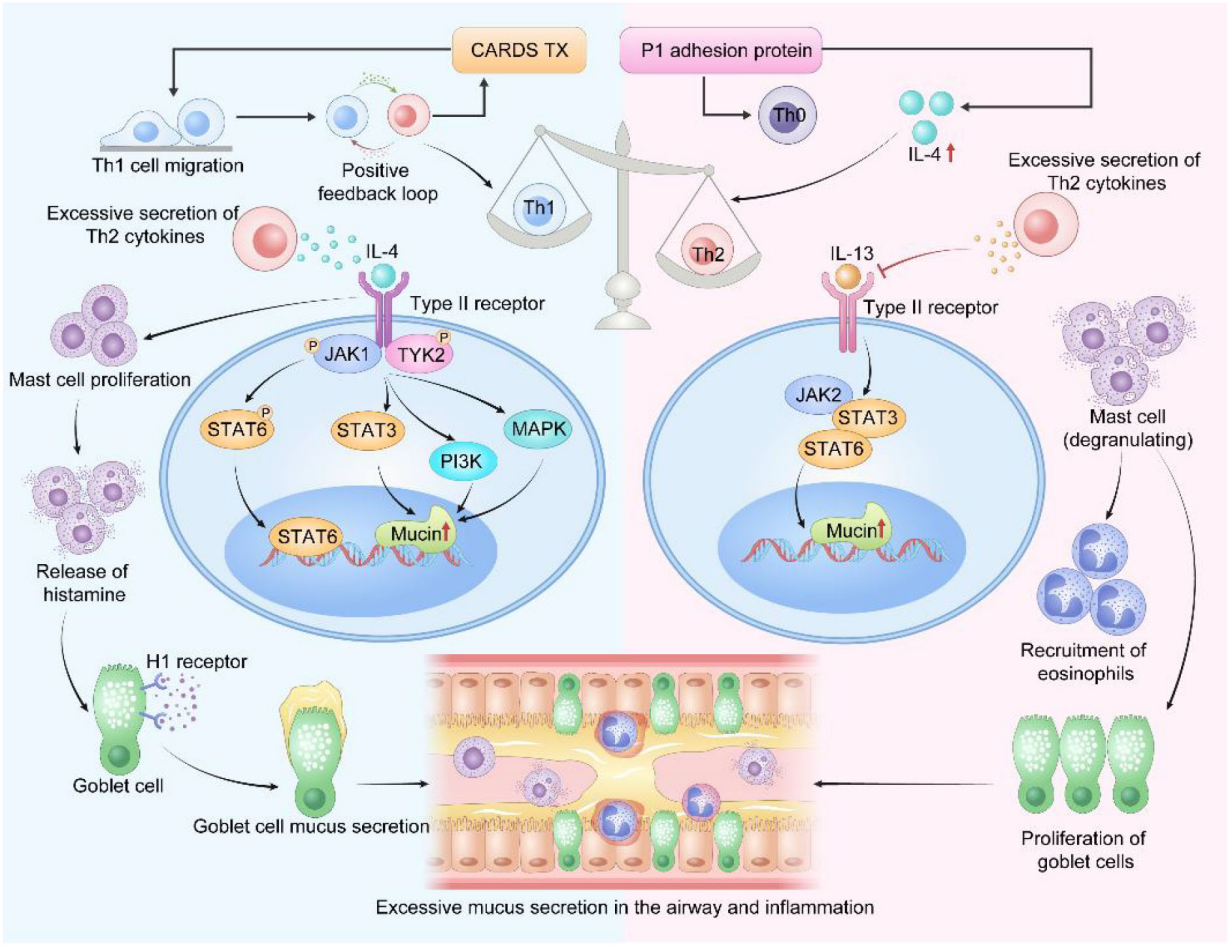
Imbalance of Th1/Th2 Cytokines. CARDS Tx produced by MP activates a positive feedback loop of type 1 immune response, promotes Th1 cell migration, and leads to Th1/Th2 imbalance, resulting in a significantly decreased ratio. This imbalance induces excessive secretion of Th2 cytokines, primarily involving IL-4 and IL-13. (Adobe Illustrator were used in the preparation of the figures).

IL-13:

IL-13 is mainly secreted by Th2 cells and mast cells, and exerts multiple roles in airway pathophysiological processes, including promoting mucus secretion, inducing goblet cell metaplasia and proliferation, and stimulating smooth muscle contraction leading to airway remodeling. IL-13 can inhibit the activation of Th1 cells, resulting in Th1/Th2 imbalance and further promoting excessive secretion of Th2 cytokines (Iwaszko et al., 2021; Leyva-Castillo et al., 2021). IL-13 shares the type II receptor system with IL-4 and promotes mucus hypersecretion by activating key signaling molecules such as JAK-STAT, PI3K, and MAPK (Walford and Doherty, 2013; Iwaszko et al., 2021). Activated STAT6 translocates into the nucleus, where it upregulates MUC5AC expression while concurrently suppressing the DNA-binding activity of forkhead box protein A2 (FOXA2) (Jung et al., 2024). FOXA2 is a critical transcription factor for maintaining normal differentiation of airway epithelial cells and suppressing goblet cell metaplasia and mucin gene expression. Thus, through the dual-pronged drive of STAT6 activation and FOXA2 inhibition, IL-13 potently induces mucus hypersecretion. A study by A. J. Myung demonstrated that inhibition of IL-13 significantly reduces the activation of the JAK2/STAT3/6 pathway, thereby effectively suppressing airway mucin production. Rho kinase inhibitor alleviates mucus hypersecretion and inflammation via downregulation of STAT6 and NF-κB (Xie et al., 2015; Jung et al., 2023). IL-13 can act back on mast cells, promoting mast cell degranulation and the release of inflammatory mediators such as histamine. It facilitates airway inflammation by recruiting eosinophils, and induces airway hyperresponsiveness and mucus hypersecretion by stimulating goblet cell proliferation (Husna et al., 2022) (**Figure 3**).

#### 4.2.4 Other cytokines mediate mucus hypersecretion following MP infection

IL-8:

MP infection can induce the activation of macrophages and epithelial cells to produce IL-8. A large amount of IL-8 can be detected in BALF during the acute phase of MP infection, and elevated IL-8 levels are closely associated with mucus retention (Chen et al., 2016; Chen et al., 2023). Studies have shown that IL-8 can directly upregulate the expression of airway mucin genes MUC5AC and MUC5B. It regulates mucin synthesis at the post-transcriptional level by binding to RNA-binding proteins in the 3’-untranslated region (3’-UTR) of MUC5AC transcripts, thereby extending mRNA half-life (Bautista et al., 2009). Additionally, IL-8 is a potent neutrophil activator that can further enhance neutrophil activity, promoting airway mucin secretion through the MP/IL-8/neutrophil axis. Furthermore, the activation of NF-κB and MAPK can also promote IL-8 production (Chmura et al., 2008; Chen et al., 2016).

IL-10:

IL-10 is a pleiotropic anti-inflammatory cytokine predominantly produced by Treg cells, macrophages, and B cells. In immune responses, it exerts inhibitory effects on the release of pro-inflammatory cytokines including TNF-α and IL-6, suppresses excessively activated inflammatory reactions, and plays a pivotal role in fostering an anti-inflammatory milieu by upregulating IL-1Ra (Reddy et al., 2024). Serum IL-10 levels in patients with severe Mycoplasma pneumoniae pneumonia (SMPP) are significantly lower than those in patients with mild disease, indicating that IL-10 insufficiency may contribute to the exacerbation of inflammatory responses (Ding et al., 2016). Studies have demonstrated a close association between IL-10 deficiency or impaired function and the formation of airway mucus plugs. Conversely, research has validated that increased IL-10 levels are correlated with the inhibition of the EGFR and MAPK signaling pathways, which directly modulates the expression of mucins MUC5AC and MUC5B (Xu et al., 2023).

IFN-γ:

IFN-γ is primarily produced by T lymphocytes, macrophages, mucosal epithelial cells, and NK cells, and participates in immune regulation by activating the JAK-STAT signaling pathway (Ding et al., 2022). It plays a crucial role in respiratory infectious diseases. Clinical data have shown that IFN-γ levels are significantly elevated in patients with MP infection, and the degree of elevation is positively correlated with the severity of infection (Wang et al., 2022; Deng et al., 2023). Studies have indicated that IFN-γ exerts an important regulatory effect on airway mucus secretion. A study by Na Lin et al. reported findings inconsistent with clinical data, demonstrating that decreased IFN-γ levels can promote the production of MUC5AC (Lin et al., 2024). This discovery is consistent with the research results of Takahito Oyanagi (Oyanagi et al., 2017), who confirmed that IFN-γ can significantly inhibit the expression of MUC5AC. The underlying mechanism may be related to the inhibition of JAK1/2-STAT6 signaling pathway activation, which further reduces airway mucus secretion and MUC5AC protein expression (Xu et al., 2023).

TNF-α:

Airway epithelial cells form the first line of defense against MP infection by secreting inflammatory mediators such as TNF-α. This cytokine not only recruits inflammatory cells but also induces local inflammatory responses, airway remodeling, airflow limitation, and impaired lung function (Chen et al., 2022). Clinical studies have shown that TNF-α levels are significantly elevated following MP infection, which activates NF-κB and MyD88. Activation of NF-κB can significantly promote the gene expression of airway mucin MUC5AC (Kurakula et al., 2015; Xie et al., 2015; Poachanukoon et al., 2017; Chen et al., 2022; He et al., 2024). MyD88 primarily activates immune responses through the HMGB1-TLRs-MyD88 signaling axis. MP infection can induce high expression of HMGB1, which is associated with the severity of infection, and HMGB1 is significantly elevated in patients with RMPP (Ding et al., 2018). HMGB1 further activates the HMGB1-TLRs-MyD88 signaling axis to increase the secretion of MUC5AC and MUC5B (Kummarapurugu et al., 2018).

TGF-β:

MP infection can significantly induce the production of TGF-β and promote airway mucus secretion, a process closely associated with immune dysregulation and inflammatory responses in the host. Studies have shown that serum TGF-β levels are significantly elevated in children with MP infection, particularly in those with wheezing symptoms (Harrop et al., 2013; Dickinson et al., 2019; Jiang et al., 2022). MP infection upregulates TGF-β expression; among its isoforms, TGF-β3 can increase ROS production via a NOX4-dependent pathway in allergic inflammation models, thereby activating autophagy and upregulating MUC5AC expression (Zhang et al., 2019). Additionally, research has demonstrated that inhibiting TGF-β can suppress Th2 cell differentiation and the release of cytokines such as IL-4 and IL-13, thereby reducing IgE antibody production. Ultimately, this inhibits mast cell degranulation and histamine release, leading to decreased airway mucus formation (Rao et al., 2025).

#### 4.2.5 Regulatory mechanisms of signaling pathways

MP infection activates multiple intracellular signaling pathways, which collectively regulate mucin gene expression and mucus secretion. Among these, the NF-κB and MAPK pathways are recognized as key regulatory pathways.

NF-κB:

NF-κB is a pleiotropic transcription factor that plays a pivotal role in regulating immune responses. Cytokines such as TNF-α produced following MP infection activate NF-κB. Prior to activation, NF-κB subunits are sequestered in the cytoplasm by IκB proteins. Upon activation, IκB-α is phosphorylated by IKK2, leading to IκB protein degradation and NF-κB nuclear translocation. Activation of NF-κB in the nucleus can directly upregulate MUC5AC gene expression, while its inhibition reduces mucin secretion (Fujisawa et al., 2011; Seo et al., 2014; Kurakula et al., 2015; Xie et al., 2015; Poachanukoon et al., 2017; He et al., 2024). Additionally, activated NF-κB can promote the release of cytokines such as TNF-α, forming a positive feedback loop that further stimulates mucus secretion (Liu Y. et al., 2020).

MAPK:

MAPK acts synergistically with IL-13 to participate in the process of airway mucin production. MAPK is activated in respiratory diseases and is essential for mucus generation. Studies have shown that MAPK13–14 are involved in the main processes of airway inflammation and mucus hypersecretion, while inhibition of the MAPK13–14 pathway can significantly reduce IL-13-induced MUC5AC expression (Keeler et al., 2023). Research has demonstrated that the p38 MAPK pathway, a subset of MAPK, also contributes to airway mucin production. IL-13 activates p38 MAPK through STAT6-dependent *de novo* protein synthesis, thereby regulating mucus cell metaplasia. Additionally, studies have indicated that the MAPK pathway can further activate NF-κB (Yang and Yu, 2021), providing a theoretical basis for the crosstalk mechanism among multiple pathways following MP infection. This crosstalk amplifies mucus secretion signals, leading to a persistent state of mucus hypersecretion.

**Figure 4:**
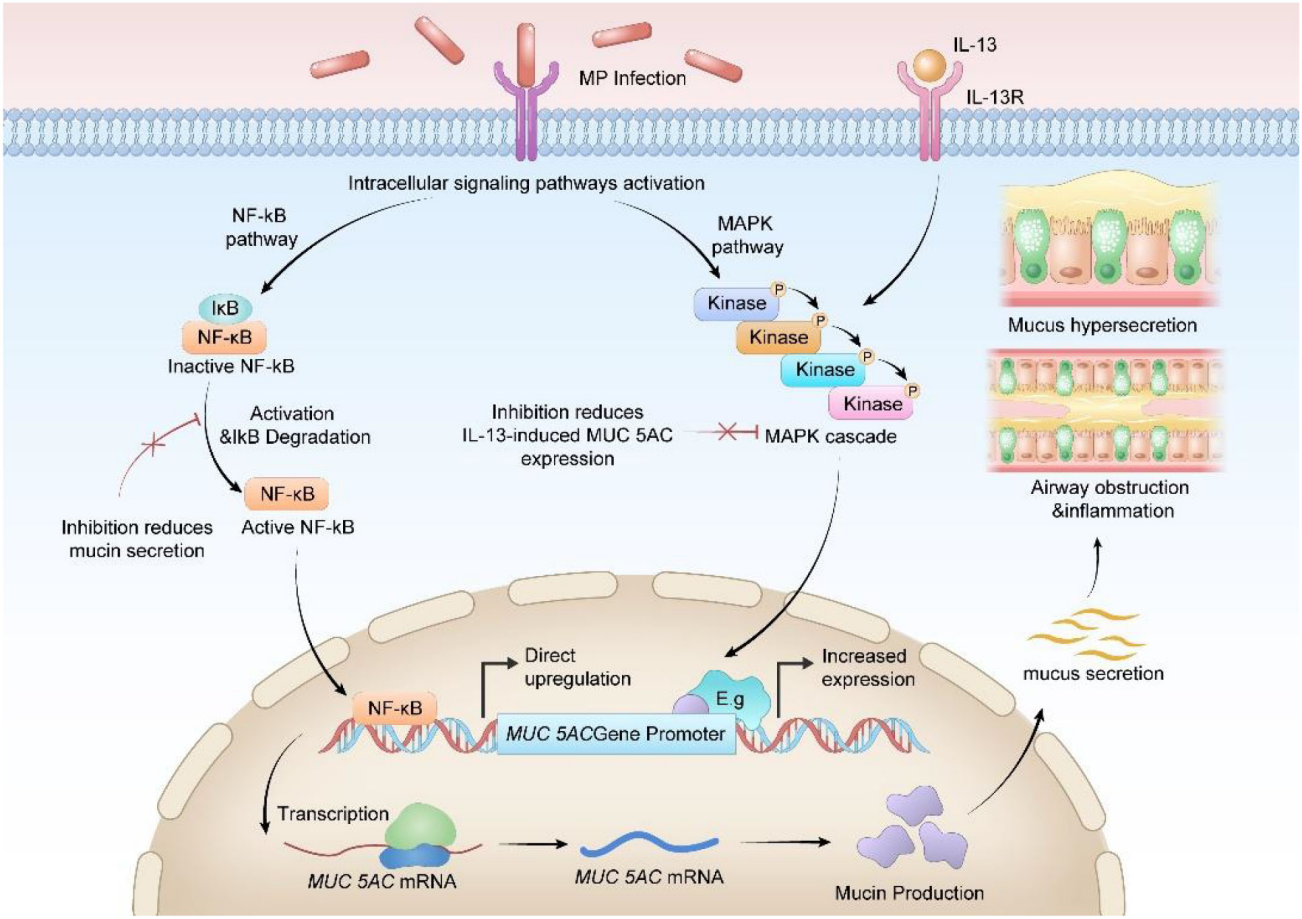
MP infection activates multiple intracellular signaling pathways, which collectively regulate mucin gene expression and mucus secretion. Activation of NF-κB in the nucleus directly upregulates MUC5AC gene expression, while inhibition of NF-κB leads to a reduction in mucin secretion. MAPK, in synergy with IL-13, participates in the process of airway mucin production. (Adobe Illustrator were used in the preparation of the figures).

EGFR:

The EGFR pathway also plays an important role in MP-induced mucus secretion. Studies have found that the CARDS Tx produced by MP can activate a positive feedback loop of type 1 immune responses, promote Th1 cell migration, and lead to an imbalance in the Th1/Th2 ratio, which is significantly lower than the normal level (Parrott et al., 2016; Yang et al., 2019; Wang et al., 2022). Th2 cytokines can amplify mucus-induced EGFR signaling. Bronchial epithelial cells and immune cells produce ligands that induce downstream EGFR signals, namely the cRAF-MEK-ERK and PI3K-AKT signaling cascades, in an autocrine manner, thereby inhibiting FOXA2 and increasing the production of MUC5AC and MUC5B (Choi et al., 2020). Activation of EGFR can further promote bronchial epithelial cells to produce IL-8 (Arae et al., 2011), resulting in airway mucus hypersecretion. In other diseases, crosstalk exists between the EGFR signaling pathway and the NF-κB pathway (Li et al., 2024), which may suggest that an EGFR-NF-κB positive feedback loop can be further formed after MP infection, thereby amplifying mucus secretion signals (**Figure 4**).

### 4.3 Changes in mucus properties leading to the formation of mucus retention

#### 4.3.1 Airway surface dehydration

Airway mucus is composed of 97.5% water (Hill et al., 2022), and changes in its hydration state play a key role in mucus retention. Dysfunction of the cystic fibrosis transmembrane conductance regulator (CFTR) provides a typical example: CFTR gene mutations can lead to congenital mucus clearance disorders, with mechanisms involving: 1) Airway surface dehydration causes abnormal increases in the concentrations of mucins, especially MUC5AC and MUC5B; 2) The osmotic pressure of the mucus layer exceeds that of the periciliary layer, creating an osmotic gradient; 3) Progressive ciliary compression and impaired transport function; 4) Ultimately resulting in abnormal mucus retention (Zhou-Suckow et al., 2017). Although the association between MP infection and abnormal CFTR gene expression has not been clearly established, considering that changes in mucus rheological properties are a typical feature of MP pneumonia and that mucus retention requires a precise balance between mucin concentration and hydration, exploring the potential impact of MP infection on CFTR expression may provide a new research direction for elucidating the mechanism of infectious mucus retention. Validation of this scientific hypothesis will help develop targeted therapeutic strategies for infectious mucus retention.

#### 4.3.2 Changes in mucin properties induced by the internal environment

As the major mucin components in airway mucus, MUC5AC and MUC5B exhibit distinctly different molecular structural characteristics. MUC5AC forms compact dimers and higher-order polymers via N-terminal disulfide bonds, and this branched cross-linked structure is closely associated with its role in mucus gel formation (Carpenter et al., 2021). In contrast, MUC5B forms linear polymers through interactions between N-N and C-C terminal disulfide bonds, presenting a hair-like bundled structure; this conformation endows it with strong resistance to proteolysis and partial reduction (Ridley et al., 2019; Carpenter et al., 2021).

MP infection can alter the physicochemical properties of mucins through multiple pathways, primarily involving internal environment disturbance. Clinical observations have shown that MP infection can induce acidosis and ionic disorders, and studies have confirmed that the activity of its CARDS Tx is pH-dependent (Ramasamy et al., 2021). Under acidic conditions: the calcium-binding sites at the N-terminus of mucins are exposed, promoting protein cross-linking/polymerization (Carpenter et al., 2021); the C-terminal dimerization domain interacts with the polymerization domain, leading to molecular compaction (Ridley et al., 2019);the molecular conformation transforms from isotropic random coils to anisotropic extended coils. Ambient temperature not only indirectly regulates mucin function by affecting pH stability but may also directly alter its molecular conformation (Mystkowska et al., 2020). These findings provide a new perspective for understanding the molecular mechanism of mucus retention after MP infection, particularly revealing the potential mechanism by which pH and temperature promote pathological changes by altering the higher-order structure of mucins.

#### 4.3.3 Peroxidase-mediated mucin cross-linking

The mechanism of mucus retention involves IL-13-mediated pathological processes. Under normal conditions, mucus secreted by human airway epithelial cells is in a liquid state; however, upon IL-13 activation, enhanced peroxidase activity can induce cross-linking of mucins in mucus, leading to a solid-like transformation (Liegeois et al., 2024). Oxidative damage is one of the important pathogenic mechanisms of MP. Although MP itself lacks peroxidase, the superoxide free radicals it produces can inhibit the activity of host cell peroxidase (Hu et al., 2022). Notably, MP infection may activate human airway epithelial cells by promoting IL-13 production. This mechanism may compensate for the lack of endogenous peroxidase in MP, thereby facilitating the solidification of airway mucins and ultimately leading to mucus retention.

#### 4.3.4 Impairment of MCC

**Figure 5:**
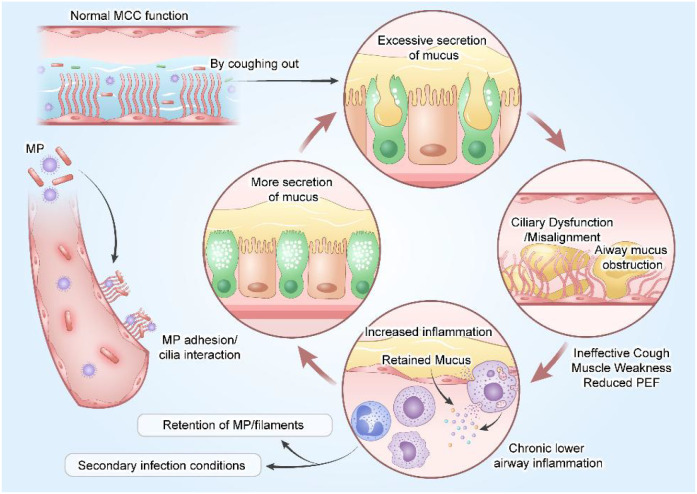
Weakened mucociliary clearance (MCC) function leads to increased mucus secretion, thereby causing airway mucus obstruction and blockage. MP adheres to the bronchial ciliated epithelial surface via adhesion and interacts with the host respiratory epithelium, further resulting in disordered arrangement and significant misalignment of adjacent cilia. Impaired ciliary function, distal airway obstruction, and ineffective coughing due to respiratory muscle weakness and reduced peak expiratory flow together complicate the mucus clearance process. Meanwhile, impaired mucociliary clearance leads to the retention of microparticles (MP) and their fragments in the airways, triggering chronic lower airway inflammation, compromised mucociliary clearance, and increased mucus secretion—these factors collectively create conditions for the persistence of particles or secondary infection. (Adobe Illustrator were used in the preparation of the figures).

The MCC system provides an important protective function for the airways. The rhythmic beating of cilia transports mucus, fluids, inhaled particles, pathogens, and dissolved chemicals from the distal airways to the proximal airways, which are then expelled from the body through coughing. Reduced MCC leads to increased mucus secretion, resulting in airway mucus obstruction and blockage (Zhou-Suckow et al., 2017). MP adheres to the surface of bronchial ciliated epithelium through adhesion and interacts with the host respiratory epithelium (Hu et al., 2022), further causing disarray and significant misalignment of adjacent cilia. This indicates that ciliary beating may lose synchronization, suggesting ciliary dysfunction (Carson et al., 1980; Osadnik et al., 2014). Poor ciliary function, distal airway obstruction, and ineffective coughing secondary to respiratory muscle weakness and reduced peak expiratory flow complicate mucus clearance. Meanwhile, impaired MCC leads to the retention of MP and its fragments in the airways, inducing chronic lower airway inflammation, impaired mucociliary clearance, and increased mucus production—creating conditions for the persistent presence of MP or secondary infections. Retained mucus serves as a hotbed for inflammatory cell infiltration, activation, and mediator release, forming a vicious cycle of “mucus hypersecretion - clearance impairment - exacerbated inflammation - more mucus secretion” (Guilbert and Denlinger, 2014) (**Figure 5**).

## 5 Treatment of mucus retention following MP infection

### 5.1 Pharmacological treatment

#### 5.1.1 Macrolide antibiotics

Macrolide antibiotics are the first-line drugs for the treatment of MP infection. Beyond their antibacterial effects, their role in regulating airway mucin secretion has also received widespread attention. Studies have shown that macrolide antibiotics can inhibit the production of airway mucins through multiple molecular mechanisms.

Azithromycin can reduce mucin secretion by affecting key signaling pathways. Studies by Nobuko Araki et al. have shown that azithromycin can inhibit the activation of transcription factor AP-1, thereby decreasing MUC5AC expression (Araki et al., 2010). Yoshifumi Imamura et al. further confirmed that azithromycin reduces MUC5AC production by inhibiting the phosphorylation of ERK 1/2 and IκB (Imamura et al., 2004). Additionally, research has demonstrated that azithromycin can inhibit airway mucin secretion by suppressing the activation of c-Jun, a subunit of AP-1 (Araki et al., 2010).

Clarithromycin also exhibits a regulatory effect on mucin production. Studies have shown that clarithromycin can reduce TNF-α-induced MUC5AC production by inducing MKP-1 expression and inhibiting p38 phosphorylation (Shah et al., 2017). Additionally, clarithromycin can attenuate ERK phosphorylation and suppress IL-13-mediated upregulation of MUC5AC expression (Nagashima et al., 2016).

#### 5.1.2 Mucolytic agents

N-acetyl-L-cysteine (NAC) effectively inhibits the production of MUC5AC and TNF-α by regulating the NF-κB and p38 MAPK signaling pathways (Mata et al., 2011), and is commonly administered via nebulization in clinical practice. Ambroxol inhaler exerts expectorant effects through a dual mechanism: on the one hand, it inhibits TNF-α-mediated overexpression of MUC5AC; on the other hand, it enhances mucociliary clearance function to promote sputum excretion (Zhang et al., 2016). Both drugs are effective clinical choices for the treatment of excessive airway mucus secretion.

#### 5.1.3 Anti-inflammatory agents

Among anti-inflammatory agents, glucocorticoids can inhibit the expression of NF-κB via intravenous administration, thereby reducing MUC5AC production (Ulkumen et al., 2021). Additionally, inhaled glucocorticoids [such as mometasone furoate (MF) and budesonide (BUD)] are commonly used for nebulization therapy in clinical practice. Studies have shown that these drugs can further inhibit MUC5AC protein synthesis by decreasing TNF-α levels (Poachanukoon et al., 2017).

### 5.2 Traditional Chinese medicine therapies

Traditional Chinese Medicine (TCM) has accumulated extensive clinical experience in addressing airway mucus hypersecretion and related disorders, demonstrating a systematic regulatory advantage through multi-target and multi-pathway approaches. Through therapeutic strategies such as “mucolytic,” “expectorant,” and “Xuanfei,” TCM not only alleviates clinical symptoms but also intervenes in the pathological mechanisms of mucus production at molecular and cellular levels.

A substantial body of research has demonstrated that TCM interventions can significantly inhibit pro-inflammatory cytokines closely associated with mucus secretion. Various herbal formulations have been shown to reduce levels of tumor necrosis factor-α (TNF-α), interleukin-6 (IL-6), IL-8, and IL-1β (Liu W. et al., 2020; Wei and Ma, 2025). Notably, the suppression of the IL-17 signaling pathway and Th2 cytokines (e.g., IL-4, IL-5, IL-13) represents a key mechanism through which TCM alleviates mucus hypersecretion in allergic airway diseases (Piao et al., 2021; Lu et al., 2025).

Tanreqing Injection may reduce MUC5AC expression by inhibiting inflammation-related factors and regulating the IL-17 signaling pathway (Liu W. et al., 2020). Guishao Zichuan Granules (GSZC) may inhibit the state of airway mucus hypersecretion by regulating the MUC5AC/EGFR signaling pathway (Gao et al., 2022). Additionally, Qingfei Oral Liquid (QF) can downregulate the expression of NF-κB protein, balance the levels of inflammatory cytokines, thereby alleviating airway hyperresponsiveness and airway mucin hypersecretion (Jing et al., 2020). The active components of Modified Jia Wei Su Zi Jiang qi formula may inhibit the secretion of MUC5AC by interfering with the IL-17 and NF-κB signaling pathways (Lu et al., 2025).

### 5.3 Bronchoscopic therapy

Currently, bronchoscopic interventional therapy is the primary method for removing BMPs. In children with MPP, bronchoscopy can observe airway lumen obstruction by secretions formed by mucins, and some cases show characteristic changes of PB. Through bronchoscopic procedures such as lavage, aspiration, and forceps removal, viscous secretions and branched mucus plugs can be effectively eliminated (Ma et al., 2022). In clinical practice, a comprehensive treatment regimen combining mucus plug extraction with forceps and drug lavage using BUD and NAC is commonly adopted. Studies have shown that these two drugs can inhibit the expression of airway mucins such as MUC5AC by regulating the NF-κB signaling pathway (Mata et al., 2011; Poachanukoon et al., 2017), thereby achieving the dual therapeutic goals of immediately relieving airway obstruction and continuously inhibiting excessive mucus secretion. This multimodal intervention strategy not only rapidly alleviates symptoms but also prevents mucus plug recurrence, holding significant clinical value in the treatment of MPP.

## 6 Conclusions and future perspectives

The mucus retention following MP infection is a complex, multifactorial, and multi-step pathological process. It involves multiple links, including the direct effects of MP, the release of cytokines, the activation of inflammatory signaling pathways, changes in the physicochemical properties of mucus, and impairment of clearance mechanisms. Pulmonary MP binds to respiratory epithelial cells through adhesion, releases toxins, induces goblet cells to secrete large amounts of mucins, promotes cytokine release, and activates multiple signaling pathways including NF-κB and MAPK, thereby facilitating the overexpression of mucin genes such as MUC5AC. Changes in the physicochemical properties of mucus and impairment of mucociliary clearance function disrupt the secretion-clearance balance, ultimately leading to mucus retention.

This study has several limitations. Our research primarily focuses on the mechanisms underlying airway mucus retention following acute Mycoplasma pneumoniae (MP) infection. The mechanisms of mucus retention in chronic infectious diseases such as chronic obstructive pulmonary disease (COPD) and asthma have not been thoroughly analyzed and warrant further investigation in future studies regarding the relationship between MP infection and mucus retention in chronic respiratory conditions. Additionally, the differences in immune responses between adults and children deserve in-depth exploration.

A deeper understanding of the molecular and cellular mechanisms of airway mucus retention after MP infection will not only provide a theoretical basis for clinical management but also contribute to the identification of potential therapeutic targets and the development of new treatment strategies. Building on this, future research should further investigate the pathogenic mechanisms of macrolide-resistant MP (MRMP) to mitigate its severe complications in pediatric patients. Furthermore, exploring the role of traditional Chinese medicine in treating MP-related airway mucus retention may offer novel insights for clinical practice.
